# Beneficial Effects of Epigallocatechin-3-*O*-Gallate, Chlorogenic Acid, Resveratrol, and Curcumin on Neurodegenerative Diseases

**DOI:** 10.3390/molecules26020415

**Published:** 2021-01-14

**Authors:** Ryuuta Fukutomi, Tomokazu Ohishi, Yu Koyama, Monira Pervin, Yoriyuki Nakamura, Mamoru Isemura

**Affiliations:** 1 Quality Management Division, Higuchi Inc. Minato-ku, Tokyo 108-0075, Japan; 2 Institute of Microbial Chemistry (BIKAKEN), Microbial Chemistry Research Foundation, Numazu, Shizuoka 410-0301, Japan; ohishit@bikaken.or.jp; 3 Shizuoka Eiwa Gakuin University Junior College, Suruga-ku, Shizuoka 422-8545, Japan; y.koyama@shizuoka-eiwa.ac.jp; 4 Tea Science Research Center, University of Shizuoka, Suruga-ku, Shizuoka 422-8526, Japan; monira689@yahoo.com (M.P.); yori.naka@u-shizuoka-ken.ac.jp (Y.N.)

**Keywords:** polyphenols, EGCG, chlorogenic acid, resveratrol, curcumin, NDD, ROS, AMPK, NF-κB

## Abstract

Many observational and clinical studies have shown that consumption of diets rich in plant polyphenols have beneficial effects on various diseases such as cancer, obesity, diabetes, cardiovascular diseases, and neurodegenerative diseases (NDDs). Animal and cellular studies have indicated that these polyphenolic compounds contribute to such effects. The representative polyphenols are epigallocatechin-3-*O*-gallate in tea, chlorogenic acids in coffee, resveratrol in wine, and curcumin in curry. The results of human studies have suggested the beneficial effects of consumption of these foods on NDDs including Alzheimer’s and Parkinson’s diseases, and cellular animal experiments have provided molecular basis to indicate contribution of these representative polyphenols to these effects. This article provides updated information on the effects of these foods and their polyphenols on NDDs with discussions on mechanistic aspects of their actions mainly based on the findings derived from basic experiments.

## 1. Introduction

Many observational and clinical studies have shown that consumption of diets rich in plant polyphenols have beneficial effects on various diseases such as cancer, obesity, cardiovascular diseases, diabetes, and neurodegenerative diseases (NDDs) [1,2]. Animal and cellular studies have indicated that these polyphenolic compounds contribute to such effects.

The representative polyphenols are catechins in tea, chlorogenic acids in coffee, resveratrol in wine, and curcumin (CRC) in curry turmeric made from *Curcuma longa* [1]. Among tea catechins, epigallocatechin gallate (EGCG) is a major catechin which is believed to show most profound biological effects and its major source is green tea. Black tea and oolong tea are made from the leaves of same tea plant *Camellia sinensis* as a source of green tea, but have much lower contents of EGCG due to loss occurring in production processes. There has been some confusion in the nomenclature of chlorogenic acids [3], but chlorogenic acid (CGA) in this review represents 5-caffeoylquinic acid. Wines contain *trans-* and *cis*-forms of resveratrol [4]. Since *trans*-resveratrol (presented here as RSV) has only been available commercially, most of human, animal, and cell-based experimental studies have used this form.

**Figure 1:**
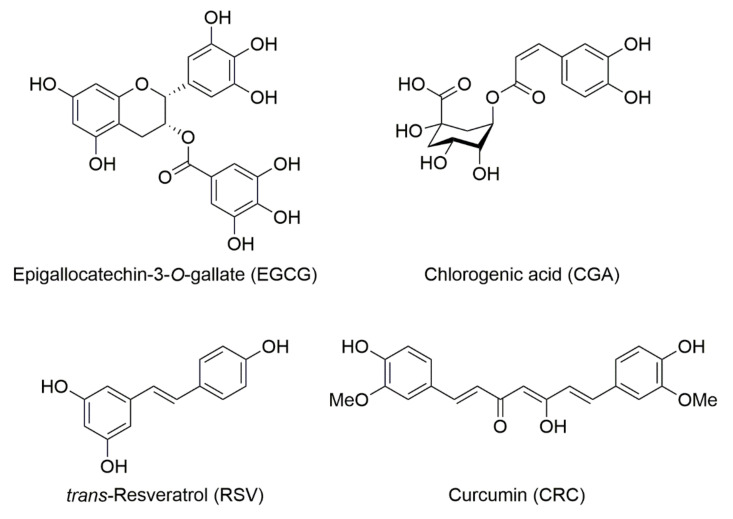
Chemical structures of epigallocatechin gallate (EGCG), chlorogenic acid (CGA), *trans*-resveratrol (RSV), and curcumin (CRC).

Chemical structures of these polyphenols are shown in Figure 1.

We have presented several review articles related to the effects of EGCG and other polyphenols on cancer, obesity, and NDDs [1,3,5,6,7]. In this article, we provide updated information on the effects of these foods and their representative polyphenols on NDDs with discussions on mechanistic aspects of their actions mainly based on laboratory findings from cellular and animal experiments.

## 2. Alzheimer’s Disease (AD)

The pathological hallmark of Alzheimer’s disease (AD) is the extracellular accumulation of amyloid plaques composed of fibrous amyloid. The proposed mechanisms for AD include microglia-triggered inflammation, over-activation of glutamate receptors, increased intracellular calcium levels, excessive generation of reactive oxygen species (ROS) and nitric oxide species, mitochondrial dysfunction, and synaptic dysfunction and loss [8]. Senile plaques are composed of β-amyloid (Aβ) peptides derived from β-amyloid precursor protein (APP) through sequential cleavages by β- and γ-secretase [9]. Accumulated deposition of the abnormal fibrous Aβ and phosphorylated tau (p-tau) proteins is one of the most characteristic features of AD that is associated with inflammation [10], elevated expression of pro-apoptotic proteins [9], and oxidative stress [11], which leads to neuronal cell dysfunction and death in the cerebral cortex [12]. Therefore, agents that suppress the formation of these biomarkers are thought to be useful for the prevention of AD [13]. APP can be cleaved by α-secretase within the Aβ domain to release soluble form of APPα (sAPPα) and preclude Aβ generation [14]. Agents that enhance α-secretase would have therapeutic potential in the treatment of AD.

### 2.1. Effects of Tea/EGCG on AD 

#### 2.1.1. Human Studies of Tea/EGCG on AD

Some studies have indicated that tea consumption is inversely associated with the risk of AD. For example, a cross-sectional survey of 2015 subjects aged 65 or older in Zhejiang province, China, found that the age-gender-standardized prevalence rates of dementia, AD, and vascular dementia were 13.0, 6.9, and 0.5%, respectively. Being elderly, low educational level, heavy smoking, heavy alcohol consumption, diabetes, and stroke were associated with dementia, whereas tea consumption was associated with a low prevalence of AD and severe cognitive impairment [15].

In contrast, several studies failed to show any beneficial effect of green tea on AD. For example, a cohort study on 2622 participants aged ≥75 with follow-up for over 10 years showed that higher green tea intake was not associated with incident AD or memory decline [16]. Kim et al. [17] conducted a meta-analysis of 20 studies that included a total of 31,479 subjects and found that caffeine intake from coffee or tea was not associated with the risk of cognitive disorders. These authors listed five different studies in which tea consumption was not associated with the risk of AD. 

While the existing evidence precludes a definite conclusion as to whether tea drinking can be preventive from AD, further research including longer-term longitudinal studies and randomized controlled trials (RCT) is warranted to shed light on this topic. Biological markers of tea consumption and AD should be employed in future research to better delineate the underlying mechanisms of tea’s benefits on cognition [18].

#### 2.1.2. Basic Studies of Tea/EGCG on AD

In an AD model mice experiment using 3% d-galactose at a dose of 150 mg/kg body weight once daily for 6 weeks, or a dose of 2 mg/kg/day or 6 mg/kg/day of EGCG for 4 weeks, EGCG supplementation significantly reduced the accumulation of Aβ and reduced neuronal injury in the hippocampus [19]. Similarly, in the TgCRND8 transgenic AD mouse model, which expresses multiple APP mutations, oral administration of EGCG at 50 mg/kg/day for 4 months exerted beneficial effects on cognition and significantly reduced soluble Aβ_1–42_ levels in the cortex and hippocampus, compared to untreated mice [20]. In an experiment using the APPSw transgenic AD mouse model, both intraperitoneally injected EGCG (20 mg/kg) and orally given EGCG (50 mg/kg) suppressed *p*-tau isoforms [21]. The finding demonstrates that EGCG downregulates tau protein, showing a potential to prevent and treat AD.

In a study using Aβ_1–42_-induced SH-SY5Y cells and APP/PS1 transgenic mice, Du et al. [22] found that EGCG prevented Aβ_1–42_-induced toxicity, increased cell viability, and inhibited neuronal apoptosis in the cortex of APP/PS1 transgenic mice, together with attenuation of endoplasmic reticulum (ER) abnormal ultrastructural swelling and downregulation of ER-stress-associated proteins. These results suggest that EGCG may attenuate the neurotoxicity in AD through inhibition of ER-stress-associated neuronal apoptosis. In a cellular experiment using neuroblastoma N2a cells, Zhang et al. [23] demonstrated that Aβ_1–42_ reduced the protein and gene expression levels of PPAR-γ coactivator-1α (PGC-1α) [23]. Overexpression of PGC-1α attenuated cell death and activation of caspase-3 induced by Aβ_1–42_ and reduced the levels of proinflammatory cytokines via inhibition of the transportation of nuclear factor (NF)-κB p65 from cytoplasm to nucleus and IκBα degradation induced by Aβ_1–42_ [23].

In a study using AD transgenic mouse model known as senescence-accelerated mouse prone 8 (SAMP8), long-term oral consumption of EGCG (15 mg/kg) improved memory function in the Y-maze and Morris water-maze tests. EGCG reduced the Aβ_1–42_ and β-secretase levels in the frontal cortex and hippocampus and prevented the hyperphosphorylation of tau [24]. Ramis et al. [25] found that old rats given (+)-catechin or Polyphenon E, a standardized GTC formulation [26] showed a significant improvement in visuo-spatial working and episodic memory. Both treatments also cancelled the age-associated reduction in the neuroinflammation via increasing sirtuin-1 (SIRT1) expression in the hippocampus. 

A recent meta-analysis of 17 preclinical studies using animal AD models found that EGCG had favorable effects in AD with shorter escape latency (standardized mean difference (SMD): −9.24, CI: −12.05, −6.42) and decreased Aβ_42_ level (standardized difference (SD): −25.74, 50% confidence interval (CI): −42.36, −9.11) [27]. These preclinical studies have proposed regulation of α-, β-, γ-secretase activity, inhibition of tau phosphorylation, anti-oxidation, anti-inflammation, anti-apoptosis, and inhibition of acetylcholinesterase (AchE) activity as the main neuroprotective mechanisms. In an experiment employing mice which received intracerebroventricular injection of 0.5 μg Aβ_1–42_, Lee et al. [28] found that EGCG dose-dependently reduced the Aβ_1–42_-induced memory dysfunction. EGCG attenuated Aβ_1–42_-induced decrease in brain α-secretase and increases in both brain β- and γ-secretase activities. In addition, EGCG was found to inhibit the activation of extracellular signal-regulated kinase and NF-κB in the Aβ_1–42_-injected mouse brains, and Aβ_1–42_-induced apoptotic neuronal cell death in the brain. Furthermore, EGCG inhibited the fibrillization of Aβ in vitro with a half maximal inhibitory concentration of 7.5 mg/L. These results suggest that EGCG’s beneficial effects on AD.

In an experiment employing human SH-SY5Y neuroblastoma and rat pheochromocytoma PC12 cells, Levites et al. [29] presented the data to show that EGCG enhanced the α-secretase-mediated release of the non-amyloidogenic sAPPα into the condition media of these cells. An inhibition or down-regulation of protein kinase C (PKC) blocked the EGCG-induced sAPPα secretion through induction of the phosphorylated PKC. EGCG also rescued PC12 cell against the Aβ toxicity. Similarly, Rezai-Zadeh et al. [30] demonstrated that EGCG promoted cleavage of the α-*C*-terminal fragment of APP to produce sAPPα along with elevated α-secretase activity and enhanced hydrolysis by tumor necrosis factor (TNF-α)-converting enzyme, a primary candidate α-secretase. These findings were confirmed in an experiment of transgenic mice over-expressing Aβ.

### 2.2. Effects of Coffee/CGA on AD

#### 2.2.1. Human Studies of Coffee/CGA on AD

There is accumulating evidence that coffee consumption may provide beneficial effects on various NDDs [31,32]. A meta-analysis of studies carried out between 1990 and 2002 allowed Quintana et al. [33] to find an approximately 28% lower risk for AD among coffee consumers compared with non-consumers. The subgroup analysis of another meta-analysis of 11 prospective studies on 29,155 participants showed a significant inverse association with a summary relative risk (RR): 0.73 (CI: 0.55, 0.97) between highest coffee consumption and the risk for AD [34]. A 5-year follow-up study with Canadian participants aged ≥65 years showed that coffee consumption, wine consumption, use of nonsteroidal anti-inflammatory drugs, and regular physical activity were associated with a reduced risk of AD [35].

A meta-analysis of prospective cohort and retrospective case-control studies on risk factors such as diet, medications, biochemical exposures, psychological condition, pre-existing disease and lifestyle in 323 papers found 4 dietary exposures (coffee, folate, vitamins C and E) as protective factors of AD [36]. A dose–response meta-analysis of 9 prospective cohort studies involving 34,282 participants found that compared with <1 cup/day, drinking of 1–2 cups/day of coffee was inversely associated with the risk of cognitive disorders (AD, dementia, cognitive decline, and cognitive impairment) with a pooled RR of 0.82 (CI: 0.71, 0.94) [37]. A clinical study with multimodal neuroimaging to examine cerebral Aβ deposition in 411 non-demented older adults found that lifetime coffee intake of ≥2 cups/day was significantly associated with a lower Aβ positivity compared to coffee intake of <2 cups/day, suggesting that higher coffee consumption may contribute to reduction of the risk of AD or related cognitive decline by decreasing pathological cerebral amyloid deposition [38].

Oppositely, several human studies did not show beneficial effects of coffee on AD. An examination for dementia in 1991-1993 showed no significant associations between coffee or caffeine intake and risk of AD, cognitive impairment, overall dementia, vascular dementia, or moderate/high levels of the individual neuropathologic lesion types, although higher caffeine intake was associated with lower odds of having any of the lesion types at autopsy [39]. A meta-analysis of 5 studies revealed no association between coffee consumption and AD. The RR of AD per 1 cup/day increment of coffee consumption was 1.01 (CI: 0.95, 1.07) [40]. Two-sample Mendelian randomization applied to large case-control and cross-sectional studies suggested that coffee may not have beneficial effects on AD, depression or type 2 diabetes [41]. 

#### 2.2.2. Basic Studies of Coffee/CGA on AD

Ishida et al. [42] investigated the effect of CGAs on the prevention of cognitive dysfunction in APP/PS2 transgenic mouse model of AD in which animals received either a control or a CGA diet. The results indicated that chronic ingestion of CGA ameliorated cognitive deficits and prevented Aβ deposition and neuronal loss in these mice. CGA enhanced the gene expression of hippocampal low-density lipoprotein (LDL) receptor-related protein 1, which has a key role for Aβ clearance and cognitive function maintenance, and restored the perivascular localization of aquaporin 4, which facilitates Aβ clearance [43]. In a Aβ-induced cell model experiments, Shi et al. [44] found that CGA increased the viability and decreased apoptosis of hippocampal neurons from newborn Sprague–Dawley rats treated with Aβ_25-35_. CGA decreased activities of lactate dehydrogenase and the malondialdehyde (MDA) levels, and raised contents of superoxide dismutase (SOD) and glutathione peroxidase (GSH)-Px in Aβ_25-35_-treated cells, suggesting that CGA restrained the apoptosis of Aβ_25-35_-induced hippocampal neurons by improving the anti-oxidant capacity, mitochondrial injury, and the state of ER stress in cells.

A study using an experimental protocol that combines NMR spectroscopy and atomic force microscopy showed that green and roasted coffee extracts, CGA, and melanoidins can inhibit Aβ aggregation and toxicity in human neuroblastoma SH-SY5Y cells [45]. Han et al. [46] found that when SH-SY5Y cells were incubated with 10 μM Aβ along with 20 μM CGA, the cells became more viable compared to SH-SY5Y cells incubated without CGA. The results of an animal experiment showed that the administration of CGA improved spatial learning and memory in SAMP8 mice, which are senescence-accelerated-prone mice that exhibit age-related deterioration in learning and memory having plaques resembling AD like depositions of Aβ. 

Another study conducted by Oboh et al. [47] demonstrated that CGA inhibited AChE activity in rat brain homogenates in a dose-dependent manner, suggesting its beneficial effect on AD, since inhibition of AChE represents the primary treatment modality against the cognitive impairment observed in AD [48]. Molecular docking studies revealed that CGA has the most significant binding affinity towards AChE [49]. As an additional model of learning and memory impairment like AD, scopolamine has been used to induce cognitive impairment in rodents [50]. Scopolamine is a muscarinic receptor antagonist that elevates oxidative stress in the brain by inhibiting ATPase and significantly increasing AChE and MDA levels in the hippocampus and cortex [51]. Kwon et al. [50] demonstrated that CGA improved the impairment of short-term or working memory and cognitive impairments induced by scopolamine. CGA inhibited AChE and decreased MDA levels in the hippocampus and frontal cortex. 

In an experiment using SH-SY5Y cells, Fukuyama et al. [52] found that roasted coffee reduced Aβ accumulation in culture medium and β-secretase expression to 70% of control levels at 12 h-incubation. Coffee activated cAMP-dependent protein kinase and pyrocatechol, a product from CGA during roasting, also reduced α-secretase expression with activation of proteasomal activity and Aβ production in these cells. These results suggest that the roasted coffee may be useful for the protection of AD. Peroxisome proliferation-activated receptor (PPAR)-α activates gene coding of α-secretase, which is responsible for non-amyloidogenic pathway of APP degradation and downregulates β-secretase, the main enzyme responsible for Aβ peptide release in AD. A recent study showed that gene expression of PPAR-α and PGC-1α was decreased in AD [53]. Since CGA significantly elevated the expression level of mRNA and protein expression in hepatic PPAR-α [54], one can suggest CRC’s upregulation of α-secretase and downregulation of β-secretase, leading to AD prevention.

### 2.3. Human Studies of Wine/RSV 

#### 2.3.1. Human Studies of Wine/RSV on AD 

Several studies have found beneficial effects of wine drinking on NDDs such as incident dementia and AD. A study on 3777 subjects aged 65 years found that the ORs in the moderate drinkers consuming 3 to 4 standard glasses per day (>250 and up to 500 mL) were 0.19 for incident dementia and 0.28 for AD after adjusting for possible confounders [55]. For the 922 mild drinkers (<1 to 2 glasses per day), there was an inverse association only with AD. In a study to examine whether intake of a single food such as red wine, white wine, coffee, green tea, and olive oil is associated with either incident AD or verbal memory decline, follow-up for over 10 years found that only higher red wine intake was associated with a reduced risk for AD but only in men [16]. Women could be more susceptible to detrimental effects of alcohol.

In a clinical study, mild-moderate AD subjects were treated with RSV or placebo for 52 weeks [56]. The results showed that compared to the placebo group, RSV markedly reduced matrix metalloproteinase (MMP)-9 in cerebrospinal fluid (CSF) and increased macrophage-derived chemokine, IL-4, and fibroblast growth factor-2. In the subset analysis, RSV attenuated declines in cognitive test scores and CSF Aβ_42_ levels, but did not alter tau protein levels. The RSV-induced decrease in MMP-9 levels may contribute to reducing inflammation and maintenance of blood-brain barrier, leading to prevention of the infiltration of leukocytes into the brain.

#### 2.3.2. Basic Studies of Wine/RSV on AD 

A number of animal and cell-based experiments have revealed beneficial effects of RSV on NDDs including AD. Freyssin et al. [57] summarized 9 studies using murine AD models to show RSV’s effects. For example, in a study conducted by Vingtdeux et al. [58] using APP/PS1 transgenic mice, a mouse model of cerebral amyloid deposition, diet supplementation of RSV during 15 weeks resulted in a reduction of Aβ levels and amyloid deposition in the cerebral cortex possibly through inhibition of mammalian target of rapamycin to trigger autophagy and lysosomal degradation of Aβ. These authors also demonstrated that orally administered RSV was detected in the brain and caused an increase in cytosolic calcium to promote calmodulin-dependent protein kinase kinase-β (CaMKK-β)-dependent activation of 5′-AMP-activated protein kinase (AMPK). 

In the SAMP 8 mice, a model of accelerated aging and age-related AD, supplementation of RSV (1 g/kg) extended the average life expectancy, activated AMPK and pro-survival pathways such as SIRT1, and reduced cognitive deficiency [59]. Other favorable effects include decreases in tau hyperphosphorylation, TNF-α levels, and NF-κB expression, and increases in glycogen synthase kinase (GSK)-3-β phosphorylation and microglial activation [57]. These authors have proposed that the neuroprotective effects of RSV are mainly due to its capacity of: (1) activation of the signaling pathways mediated by AMP-activated protein kinase (AMPK), phosphoinositide 3-kinase and Akt; (2) promotion of synaptic plasticity by extracellular signal-regulated kinase (ERK) 1/2; (3) inhibition of pathways involved in apoptosis; (4) reduction of amyloidogenesis; (5) enhancement of the clearance of Aβ; (6) radical scavenging; and (7) suppressing inflammation [57,60]. 

Soo et al. [61] summarized effects of RSV on cultured cells and animal models of *C. elegans*, *Drosophila*, and mice/rats. RSV showed protective effects on AD in 6 cellular studies and 8 animal studies. In cell-based studies, RSV significantly reduced the cytotoxicity of Aβ_1–42_ peptide in SH-SY5Y human neuroblastoma cells, suggesting a protective effect of RSV. Atomic force microscopy analyses revealed that Aβ_1–42_ peptide aggregated spontaneously into oligomers, protofibrils, and fibrils, but RSV caused a distinct lack of these structures, indicating fragmentation of Aβ_1–42_ into smaller peptides, which have no propensity to aggregate further [62]. This finding provides additional evidence to show the RSV’s role to previously reported inhibitory activities against β-secretase and expression of APP [63,64].

The results of an experiment, in which 6-month-old male PS19 mice were treated with RSV or vehicle by oral administration once a day for 5 weeks, revealed that RSV rescued cognitive impairments and reduced the levels of p-tau, neuroinflammation, and synapse loss in the brains of mice [65]. RSV also reduced the TNF-α and IL-1β levels in the brain lysates of these mice. In a cellular study using N2a cells, RSV was shown to inhibit tau protein aggregation and tau oligomer-induced cytotoxicity, and blocked the uptake of extracellular tau oligomers by these cells [65]. 

Telomere shortening plays an important role in AD [66]. Granzotto and Zatta [67] pointed out that RSV promotes the expression of Werner syndrome ATP-dependent heicase, a telomere maintenance factor, increases the activity of telomerase through an SIRT1-dependent pathway, and protects telomeres and DNA from ROS-dependent damages [68,69,70].

### 2.4. Studies of Curry/CRC on AD

#### 2.4.1. Human Studies of Curry/CRC on AD

Some human studies have suggested the beneficial effects of curry/CRC on AD. An Indo-US cross-national dementia study reported a 4.4-fold less prevalence of AD in the 70–79 age group in India than in the US. The regular intake of turmeric as curry spice in the common Indian diet has been considered as the primary reason for this epidemiological observation [71,72]. In a case report of 3 AD patients who exhibited irritability, agitation, anxiety, and apathy, Hishikawa et al. [73] described that supplementation of turmeric powder for 12 weeks improved a mental state examination score from 12/30 to 17/30 in one patient. These participants could recognize their family within 1 year. These findings suggest that turmeric is useful to improve the behavioral symptoms in AD. 

DiSilvestro et al. [74] evaluated CRC’s effects under normal physiological conditions. In a placebo-controlled study, 38 healthy middle-aged subjects consumed orally 80 mg/day of CRC in a lipidated form for 4 weeks. The results showed that CRC, but not placebo, caused the significant changes including decreases in plasma Aβ concentrations, plasma triglyceride values, salivary amylase levels, and plasma alanine aminotransferase activities, and increases in salivary radical scavenging capacities, plasma catalase and plasma myeloperoxidase activities, and plasma nitric oxide levels. These results suggest that CRC can exhibit a variety of health promoting effects including anti-AD effect. 

In contrast, several studies failed to show CRC’s benefits. For example, an RCT to evaluate the efficacy of two dosages of CRC (2 and 4 g/day) in patients with mild-to-moderate AD showed no significant differences in cognitive function, in plasma or CSF Aβ_40_/Aβ_42_ or tau, between placebo and intervention groups, perhaps due to low bioavailability of CRC [75]. A clinical study in which 34 AD patients were randomized to receive 1 g (plus 3 g placebo), 4 g (plus 3 g placebo), or 0 g of oral CRC (plus 4 g of placebo) showed that the intervention group did not demonstrate significant differences in cognition examination scores or plasma Aβ_40_ levels between 0 and 6 months [76]. It was suggested that the outcome measures were not sensitive or specific enough to demonstrate effects.

In an RCT in which 36 subjects with mild-to-moderate AD were randomized to receive 2 g/day or 4 g/day of oral CRC formulation or placebo, Ringman et al. [75] found no differences between these groups in clinical or biomarker efficacy measures including the levels of Aβ_1–40_ and Aβ_1–42_ in plasma and those of Aβ_1–42_, t-tau, and p-tau181 in CSF (cerebrospinal fluid). In a recent review, Bhat et al. [77] summarizes 22 clinical trials on CRC and its formulations for different neurodegenerative diseases and patent details in India [77]. This survey did not find any report that had confirmed or claimed the neurotoxic effect of CRC. Further studies may provide benefits of CRC in AD patients.

#### 2.4.2. Basic Studies of CRC on AD and Action Mechanism 

A large number of basic studies have examined CRC’s effects on AD. In a recent review, Voulgaropoulou et al. [78] included 21 animal studies to show CRC’s favorable effects on AD.

The most promising application of CRC in NDD therapy may be its anti-amyloid property [79]. CRC binds not only Aβ-oligomers and fibrils in AD complications [80], but also α-synuclein in PD [81] and p-tau found in tauopathies and AD [82]. Ono et al. [83] demonstrated that CRC had dose-dependent effects on the inhibition of Aβ_1–40_/_1–42_ fibrils, with an EC_50_ of 0.09–0.63 µM. Several in vitro studies demonstrated that CRC can attenuate the assembly of both Aβ_40_ and Aβ_42_ oligomers and fibril formation [84]. In an animal model of AD, after oral or intraperitoneal injections of CRC for 3–7 days in mice, CRC was detected in brain tissue with decreasing neuropathology, indicating its passage through the blood-brain barrier (BBB) [85]. Similarly, significant inhibition of Aβ oligomerization, its plaque formation, and tau phosphorylation, along with behavioral improvements were observed in a mouse model of AD after oral administration of CRC [79,86]. A multiphoton microscope examination revealed a decrease in 30% Aβ plaque size and prevention of dystrophic neurites after tail vein injection of CRC [85]. 

CRC may inhibit the activity of β-secretase to reduce the levels of Aβ [79,86] and the APP metabolic pathway to lower Aβ levels [84], and also regulate Aβ production by inhibiting GSK-3β-mediated presenilin-1 activation [87]. An AD model mouse experiment revealed that animals received intragastric CRC administration of 150 or 300 mg/kg/day for 60 days resulted in reduction in Aβ production by downregulating β-secretase expression, prevention of synaptic degradation, and recovery of spatial learning and memory impairment [88]. An in vitro experiment showed that CRC inhibits p-tau aggregation by reducing oxidative stress [89]. CRC inhibited GSK-3β activity and reduced tau dimer and p-tau oligomerization in a human tau transgenic mouse model [90]. In addition, oral administration of CRC together with docosahexaenoic acid reduced p-tau by inhibiting insulin receptor substrate (IRS)-1 and c-Jun N-terminal kinase (JNK) activities in vivo [91]. 

CRC has potent anti-oxidant activity, which scavenges ROS, increases antioxidant levels, decreases lipid peroxidation, chelates toxic metals to reduce injury in AD brains [92,93,94] CRC was shown to downregulate the inflammatory cytokines, including IL1, IL6, TNF-α, interferon-γ, and cyclooxygenase (COX)-2 activity [95]. 

The epigenetic role of gene expression of CRC was shown by inhibiting histone acetyltransferases activity and activating histone deacetylases in AD. CRC can directly bind to histone acetyltransferase at nM levels and can inhibit its catalytic activity [96], thus inhibiting nuclear histone acetylation. Decreases in histone acetylation reduce the inflammation via NF-κB pathway in some brain diseases [97]. Recent comprehensive reviews have also discussed the animal experiments to reveal CRC’s favorable effects on murine AD models [78,98].

## 3. Parkinson’s Disease (PD)

PD is characterized by the presence of Lewy bodies mainly composed of ubiquitinated α-synuclein, neurofilaments, synaptic vesicle protein, and parkin. Lewy bodies can be involved in disorders such as the release of free radicals, excessive generation of nitric oxide species, enhanced c-Jun *N*-terminal kinase pathway activated-apoptosis, microglia-triggered inflammation, and disruption of protein degradation pathways [8]. Thus, interference with these events would help prevent neurodegenerative disease PD. 

### 3.1. Effects of Tea/EGCG on PD

#### 3.1.1. Human Studies of Effects of Tea/EGCG on PD

A recent review including prospective studies, nested case-control studies, and meta-analysis to know the relation between diet and PD risk, Boulos et al. [99] found some evidence regarding a potential protective effect of uric acid, polyunsaturated fatty acids, coffee, and tea. In fact, many epidemiological studies indicated tea’s beneficial effect on PD. In a case-controlled study with 249 PD cases and 368 control subjects, the intake of coffee, black tea, and Japanese and Chinese teas was inversely associated with the risk of PD. The adjusted odds ratio (OR)s compared with the highest to the lowest quartile were 0.52, 0.58, and 0.59, respectively (CI: 0.30, 0.90; 0.35, 0.97; and 0.35, 0.995, respectively) [100]. The result of a study on 278 PD patients showed that consumption of tea of >3 cups/day delayed onset of motor symptoms by 7.7 years [101]. The results of a pooled analysis of 11 case-control and 1 cohort studies showed that compared to non-tea consumers, tea consumers had a pooled OR of 0.73 (CI: 0.60, 0.90), indicating a clear protective effect of tea consumption in Chinese populations [102]. In a dose–response meta-analysis of a total of 13 studies on 344,895 participants, Qi and Li et al. [103] found a linear relationship between tea consumption and PD risk. The smoking-adjusted risk of PD decreased by 26% for every 2 cups/day and 200 mg/day increments. 

Checkoway et al. [104] found a reduced risk for consumption of ≥2 cups/day of tea (OR: 0.4, CI: 0.2, 0.9) as compared to almost no consumption. A study in ethnic Chinese population found dose-dependent protective effect with a 28% risk reduction of PD by one unit of coffee and tea (3 cups/day for 10 years) [105]. A follow-up of 12.9 years among 29,335 Finnish subjects aged 25–74 years showed that a multivariate-adjusted hazard ratio (HR) of PD for subjects drinking ≥3 cups/day of tea compared with nondrinkers of tea was 0.41 (CI: 0.20, 0.83) [106]. A case-control study with 75 PD patients and 75 control patients found that the control group consuming more glasses of tea than the PD group before the onset of their disease signs [107]. Every extra glass of tea per day decreased the risk of PD by 0.8 times (OR: 0.8, CI: 0.73, 0.97). 

These epidemiological findings support the beneficial effect of tea consumption on PD, but some failed to provide such evidence. For example, a cohort study conducted on 74,941 women aged 40–70 found no significant association with an age-adjusted OR of 0.8 (CI: 0.5, 1.3) for tea-drinkers with continuous drinking of at least 3 times/week for 6 months or longer [108]. Although Tan et al. [109] found an inverse association with an adjusted RR of 0.29 (CI: 0.13, 0.67) for the highest vs. lowest tertile of black tea intake, green tea was not related to PD risk in the Singapore Chinese population. Noyce et al. [110] found no evidence to support a protective effect of tea drinking, and some evidence that tea drinking may even increase the risk of PD [110]. Thus, further studies are required to assess the association between tea consumption and the risk of PD.

#### 3.1.2. Basic Studies of Effects of TEA/EGCG on PD and Action Mechanism

A number of cell-based and animal experiments have provided evidence to support that tea and green tea catechins (GTCs) have beneficial effects on brain disorders. In an PD model mice experiment using *N*-methyl-4-phenyl-1,2,3,6-tetrahydropyridine at a dose of 24 mg/kg (i.p.) for 4 days, pretreatment of mice with either green tea extract (0.5 and 1 mg/kg/day; i.p.) or EGCG (2 and 10 mg/kg) significantly protected against drug-induced dopamine decrease. In addition, oral administration of EGCG (2 and 10 mg/kg) also prevented the decrease of dopamine induced by the drug [111]. In another study using the same mouse model, administration of EGCG at 2 different doses (10 mg/kg and 50 mg/kg) reduced the drug-induced neuronal cell death rate to less than 50% [112]. In an experiment using *N*-methyl-4-phenyl-1,2,3,6-tetrahydropyridine-induced PD mouse model, Zhou et al. [113] found that EGCG treatment restored the movement behavior of the mice impaired by the drug and prevented its toxicity in tyrosine hydroxylase-positive cells in the substantia nigra pars compacta region. EGCG reduced the serum levels of inflammatory factors TNF-α and IL6. These findings indicate that EGCG exerts neuroprotective effects in the PD mice model possibly through modulating peripheral immune response.

Mutations in LRRK2 and parkin together are responsible for the majority of familial PD. Ng et al. [114] found that EGCG suppressed of dopaminergic and mitochondrial dysfunction in both mutant LRRK2 and parkin-null flies. Since loss of AMPK activity promoted neuronal loss and pharmacological or genetic activation of AMPK reproduced EGCG’s protective effects, AMPK activation may be involved in the mechanism of EGCG’s action. These studies suggest the potential use of EGCG/GTCs as a therapeutic agent for the prevention and treatment of PD.

### 3.2. Effects of Coffee/CGA on PD

#### 3.2.1. Human Studies of Effects of Coffee/CGA on PD

A number of epidemiological studies have shown the beneficial effects of coffee consumption on PD [115]. A meta-analysis of 8 case-control and 5 cohort studies showed that the RR of PD was 0.69 (CI: 0.59, 0.80) for coffee drinkers as compared with non-coffee drinkers. The RR per 3 additional cups/day was 0.75 (CI: 0.64, 0.86) in case-control studies and 0.68 (CI: 0.46, 1.00) in cohort studies [116]. In a prospective study of caffeine consumption and risk of PD, Ascherio et al. [117] found an inverse association of PD risk with consumption of coffee, caffeine from noncoffee sources or tea, but not decaffeinated coffee among men. In the case of women, the relationship between caffeine/coffee intake and risk of PD was U-shaped, with the lowest risk observed at intakes of 1–3 cups of coffee/day. These findings suggest a possible protective effect of moderate doses of coffee/caffeine on risk of PD.

Analysis of 30 years of follow-up of 8004 Japanese-American men aged 45–68 years found that age-adjusted incidence of PD decreased with increase in amounts of coffee intake, from 10.4/10,000 person-years in male nondrinkers to 1.9/10,000 person-years in male drinkers who consumed at least 28 oz coffee/day [118]. In a study using a case-control design to know the association of PD with preceding smoking, alcohol, and coffee consumption, Benedetti et al. [119] found the OR of 0.35 (CI: 0.16, 0.78) and a later age at PD onset in coffee drinkers compared with nondrinkers. The inverse association with coffee was restricted to PD cases with onset at age <72 years and to men. 

In a multivariable analysis, Gigante et al. [120] found the number of years of patients’ coffee consumption was correlated with a significant increase in age at PD onset. In a literature review to evaluate the associations between coffee and caffeine consumption and various health outcomes, evidence from meta-analyses of observational studies and RCTs suggested that coffee consumption was associated with a decreased risk of PD, several cancer types, cardiovascular disease, and type 2 diabetes [121].

A prospective study to examine the prediction of coffee consumption on the incidence of PD in 6710 men and women, aged 50–79 years with a 22-year follow-up, found that the adjusted RR for coffee drinkers of ≥10 cups/day was 0.26 (CI: 0.07, 0.99) compared with non-drinkers [122]. The result of a dose–response meta-analysis of a total of 13 articles involving 901,764 participants showed a non-linear relationship between coffee consumption and PD risk with the maximum effect at about 3 cups/day (smoking-adjusted RR: 0.72, CI: 0.65, 0.81) [103].

In spite of these positive results, some studies provided negative ones for coffee’s effect on PD. For example, a clinical survey on 1339 subjects aged ≥65 years showed that coffee and caffeine consumption was not associated with the presence of parkinsonian signs, although the odds for presence of the disease signs was lower in smokers than nonsmokers [123]. The result of a case-control study conducted in western Washington State found no associations of PD with coffee consumption or total caffeine intake or alcohol consumption, although a reduced risk was observed in consumers of ≥2 cups/day of tea (OR: 0.4, CI: 0.2, 0.9) and in smokers [104]. In addition, a case-control study involving 74 PD patients did not show an association between the risk for PD and coffee and tea drinking habits or other variables such as exposure to industrial toxins and cranial trauma [124]. 

#### 3.2.2. Basic Studies of Effects of Coffee/CGA on PD and Action Mechanism

In addition to possible beneficial effects on AD, many studies have demonstrated that CGA is a strong candidate as a preventative agent for PD [125]. Singh et al. [126] studied CGA in a drug-induced acute mouse model of sporadic PD for its antioxidant and anti-inflammatory properties and observed that CGA-treatment caused the enhanced expression of tyrosine hydroxylase within the nigrostriatal region. CGA reduced the drug-induced neuroinflammation in substantia nigra by regulating the NF-κB expression and release of certain pro-inflammatory mediators such as TNF-α and interleukin (IL)-1β with the enhanced expression of anti-inflammatory cytokine IL-10. The similar experiments also demonstrated that CGA inhibited the activation of proapoptotic proteins including B-cell lymphoma-2 (Bcl-2) associated X protein (Bax) and caspase-3 and elevated the expression of antiapoptotic protein like Bcl-2 to prevent apoptosis [127]. CGA improved phosphorylation state of Akt, ERK1/2, and GSK-3*β* which was downregulated as an effect of the drug-induced toxicity.

Teraoka et al. [125] showed that CGA attenuated a decrease in cell viability induced by α-synuclein in catecholaminergic PC12 cells and that CGA blocked the interaction of oxidized dopamine with α-synuclein via inhibition of oxidation of dopamine and the oligomerization of α-synuclein. Rotenone, a mitochondrial complex I inhibitor, is known to cause parkinsonian pathology. Miyazaki et al. [128] examined the neuroprotective effects of caffeic acid and CGA against the rotenone-induced degeneration in C57BL/6J mice. Caffeic acid and CGA prevented rotenone-induced neurodegeneration of both nigral dopaminergic and intestinal enteric neurons with upregulation of the antioxidant molecules, metallothionein 1,2 in striatal astrocytes of the model mice. 

### 3.3. Effects of Wine/RSV on PD

#### 3.3.1. Human Studies of Effects of Wine/RSV on PD

No comprehensive study has been reported on the effects of wine/RSV on PD, but some basic studies have suggested RSV’s benefits on this disease as shown below. Future human studies may reveal a merit of wine/RSV consumption for prevention of PD.

#### 3.3.2. Basic Studies of Effects of RSV on PD and Action Mechanism

In a survey of Soo et al. [61], 4 cell-based and 8 animal studies showed RSV’s beneficial effects on PD but one *Drosophila* study showed a deleterious effect. A study using cellular PD models showed that RSV enhanced autophagy through activation of AMPK/SIRT1 pathway, leading to the neuroprotection [129]. Guo et al. [130] showed that RSV attenuated drug-induced dopaminergic neurodegeneration and behavioral impairments in mice and that RSV’s activation of SIRT1 was involved in these protective effects. Activation of SIRT1 and subsequent microtubule-associated protein 1 light chain 3 caused autophagic degradation of α-synuclein, suggesting that RV may be a potential prophylactic or therapeutic agent for PD.

In an experiment using 6-hydroxydopamine-induced PD rat model, RSV alleviated drug-induced chromatin condensation, mitochondrial tumefaction, and vacuolization of dopaminergic neurons in the substantia nigra [131]. RSV also significantly reduced the levels of COX-2 and TNF-α mRNA together with the decreased levels of COX-2 protein expression in this structure [131].

### 3.4. Effects of CRC on PD

#### 3.4.1. Human Studies of Effects of CRC on PD

No appreciable data have been presented in relation to the effect of curry or CRC on PD. Some basic studies have indicated CRC’s inhibitory action on synuclein fibril formation as mentioned below so that a future study may show its beneficial effects on PD.

#### 3.4.2. Basic Studies of Effects of CRC on PD and Action Mechanism

Several studies have demonstrated CRC’s beneficial effects on PD. For example, Singh et al. [81] showed that CRC inhibited α-synuclein aggregation, prevented Lewy Bodies accumulation in vitro, and attenuated α-synuclein oligomer-induced toxicity in cells. In rotenone-induced PD model mice, CRC treatments for 3 weeks significantly improved behavioral alterations, oxidative damage, and mitochondrial enzyme complex activities as compared to the control rotenone-treated group [132]. CRC mitigated enhanced the acetylcholine esterase enzyme levels, restored motor deficits, and enhanced the activities of antioxidant enzymes suggesting CRC’s in vivo antioxidant potential. In a lipopolysaccharide (LPS)-induced animal model of PD, Sharma and Nehru [133] demonstrated that daily CRC administration for 21 days prevented the LPS-induced upregulation of the protein activity of NF-κB, proinflammatory cytokines (TNF-α, IL-1β, and IL-1α) and inducible nitric oxide synthase (iNOS). CRC regulated molecules of the intrinsic apoptotic pathway (Bax, Bcl-2, caspase 3, and caspase 9), improved the glutathione system, and prevented iron deposition in the dopaminergic neurons, and formation of α-synuclein aggregates in the dopaminergic neurons, implying CRC’s beneficial effects on PD. 

Other studies have revealed CRC’s favorable effects on PD. These include: attenuation of reduction of dopamine levels and degeneration of dopamine neurons [134]; chelation of iron, copper, and other metals to prevent α-synuclein or Lewy bodies aggregation [135]; inhibition of activity of monoamine oxidase to restore the dopamine levels [136] and reduce depression [137]; protection of dopamine neurons by reducing ROS levels, maintaining mitochondrial functions, and attenuating neuroinflammation [138]; suppression of the JNK pathway and prevention of dopaminergic neuronal loss caused by apoptosis [139].

## 4. Other NDDs and Healthy Subjects

### 4.1. Effects of Tea/EGCG

#### 4.1.1. Human Studies of Tea/EGCG on Other NDDs and Healthy Subjects

A literature review by Polito et al. [140] included 23 epidemiological studies examining association between tea intake and the risk of AD and related cognitive decline, at least 17 studies of which found favorable effects. For example, Kuriyama et al. [141] reported the results of a cross-sectional study on green tea consumption and cognitive function. The 1003 Japanese subjects aged 70 or older completed a self-administered questionnaire that included questions about the frequency of green tea consumption. The results indicated that a higher consumption of green tea was associated with a lower prevalence of cognitive impairment. After adjusting for potential confounders, ORs for cognitive impairment associated with different frequencies of green tea consumption were 1.00 (reference) for 3 or fewer cups/week, 0.62 (CI: 0.33, 1.19) for 46 cups/week or one cup/day, and 0.46 (CI: 0.30, 0.72) for ≥2 cups/day. For black or oolong tea, corresponding ORs were 1.00 (reference), 0.60 (CI: 0.35, 1.02), and 0.87 (CI: 0.55, 1.38), respectively. 

Similarly, a cross-sectional analysis of 2,501 Chinese adults aged ≥55 and a longitudinal analysis of data from 1438 cognitively intact participants found that tea intake was significantly associated with a lower prevalence of cognitive impairment. Relative to rare or no tea intake, the ORs for low, medium, and high levels of tea intake were 0.56 (CI: 0.40, 0.78), 0.45 (CI: 0.27, 0.72), and 0.37 (CI: 0.14, 0.98), respectively. For cognitive decline, the corresponding ORs were 0.74 (CI: 0.54, 1.00), 0.78 (CI: 0.55, 1.11), and 0.57 (CI: 0.32, 1.03), respectively. These effects were most evident for black and oolong teas. Although an association of green tea with less cognitive impairment was found, the association was not meaningfully separated from that due to black or oolong tea consumption due to the small number of participants who only drank green tea [142].

In a population-based prospective study with Japanese residents aged >60, the incidence of dementia during a follow-up period of about 4.9 years was 5.3%, and that of mild cognitive impairment (MCI) was 13.1%. The multiple-adjusted ORs for the incidence of overall cognitive decline was 0.32 (CI: 0.16, 0.64) among individuals who consumed green tea every day and 0.47 (CI: 0.25, 0.86) among those who consumed green tea 16 days per week compared with those who did not consume green tea. The multiple-adjusted OR for the incidence of dementia was 0.26 (CI: 0.06, 1.06) among individuals who consumed green tea every day compared with those who did not consume green tea. No association was found between coffee or black tea consumption and the incidence of dementia or MCI. These results indicate that green tea consumption is significantly associated with a reduced risk of cognitive decline [143]. 

A cross-sectional study involving 716 Chinese adults aged ≥55 in urban Singapore found that total tea consumption was independently associated with a better performance in global cognition, memory, executive function, and information processing speed. The protective effect of tea consumption on cognitive function was not limited to any particular type of tea (green tea, black tea, or oolong tea), whereas these effects were not demonstrated by coffee consumption [144]. An epidemiological study with 4579 participants of ≥60 years showed an inverse association between consumption of tea of any type and prevalence of cognitive impairment (OR: 0.74, CI 0.57, 0.98 [145]. Never smokers showed much obvious protective correlation of tea in a frequency-dependent manner. No association was found in current/former smokers [145].

An RCT conducted by Scholey et al. [146] found that 300 mg EGCG administration was associated with a significant overall increase in α-, β-, and θ-wave activities. EGCG consumption also increased self-rated calmness and reduced self-rated stress. These results suggest EGCG’s relaxing and refreshing properties, a beneficial effect of green tea intake on neuronal activity [146]. In an RCT, 23 participants consumed one of the 4 test products: matcha tea (a kind of Japanese green tea), matcha tea bar (each containing 4 g matcha tea powder), placebo tea, or placebo bar. The results indicated that consumption of matcha, compared to the placebo, significantly improved tasks that measure basic attention abilities and psychomotor speed in response to stimuli [147]. Other studies also demonstrated tea’s beneficial effects on cognitive impairment. In an RCT on adults aged 16–34 years with Down’s syndrome, de la Torre et al. [148] found that participants treated with EGCG (9 mg/kg per day) and cognitive training had significantly higher scores in visual recognition memory and adaptive behavior [148]. No differences in adverse effects were observed between the two groups. 

Conversely, some studies failed to demonstrate that green tea or EGCG significantly affected cognitive functions, although some improvements were found in physiological biomarkers of brain function. A cross-sectional study of 1143 patients with a mean subject age of 68.9 in a rural hospital in Niigata, Japan, found that age, low green tea consumption, low body mass index, a history of stroke, a history of myocardial infarction, and low fruit consumption were independently associated with a higher prevalence of cognitive impairment [149]. In an RCT in which participants were randomly allocated to the green tea or placebo group and consumed either 2 g/day of green tea powder or placebo, respectively, for 12 months, the result indicated that cognitive scores after 1 year of consumption were not significantly different from the placebo group, although levels of MDA-modified LDL, a marker of oxidative stress, was significantly lower in the green tea group [150].

A cross-sectional study among the Chinese elderly, in which 9375 residents aged ≥60 years were recruited, found that compared with non-consumption, the consumption of black tea showed a positive correlation with cognitive function after controlling for confounders (OR: 0.52, CI: 0.28, 0.95), whereas green tea showed no significant difference (OR: 1.04, CI: 0.72, 1.51) [151]. In a double-blind, placebo-controlled, crossover study, 27 healthy adults received placebo and 2 doses of EGCG. However, 135 mg and 270 mg doses of EGCG caused no changes in cognitive performance or mood, although the consumption of 135 mg EGCG reduced cerebral blood flow in the frontal cortex relative to the placebo [152].

#### 4.1.2. Basic Studies of Tea/EGCG on Other NDDs and Action Mechanism

In a comprehensive review, Singh et al. [8] summarized the cellular and animal studies on the neuroprotective effects of EGCG and GTCs. EGCG and GTCs may exert their effects by mechanisms including activity as an antioxidant (6 studies), modulation of signaling pathways (6 studies), inhibition of protein aggregation (8 studies), and modulation of apoptosis (6 studies). EGCG reduced glutamate-induced oxidative cytotoxicity in cultured mouse hippocampal neuronal HT22 cells by inactivating the NF-κB signaling pathway. Copper ions can form a high-affinity complex with Aβ, and the oxidation reactions by Cu(II)-Aβ complexes lead to the formation of Cu(I)-Aβ, which is involved in neurotoxicity via radical cation and hydroxyl radical generation [153]. The ability of EGCG to act as a chelator of transitional metals such as iron and copper cations may have significance for treatment of neurodegenerative diseases [154]. In a cell-based study, Liu et al. [155] treated macrophages with LPS to induce the expression of proinflammatory cytokines (TNF-α, IL-1β, and IL-6) and found that EGCG inhibited LPS-mediated induction of these cytokines and that a supernatant from EGCG-pretreated and LPS-activated macrophage cultures was less cytotoxic to neurons than those from non-EGCG-pretreated and LPS-activated macrophage cultures. EGCG inhibited the LPS-induced production of ROS in neurons. Thus, EGCG may exert its neuroprotective action by suppressing ROS-induced inflammation [155].

Fragile X syndrome is a leading genetic disorder of intellectual disability. De la Tore et al. [156] conducted preclinical study using an animal model and phase I clinical trial (TESXF; NCT01855971). The results showed that EGCG improved object-recognition memory in disease model mice and that EGCG combined with cognitive training improved visual episodic memory and functional competence in life of patients compared to those without combined treatment. In an experiment using one-year-old natural aging rats (400–450 g) and 6-week-old young rats (250–280 g), Wei et al. [157] found that EGCG ameliorated the cognitive impairment in the old rats with decreased Aβ_1–42_ plaque formation in the brain [157]. Orally administered EGCG was detected in the brain and the permeability of BBB was significantly increased in old rats but not in young rats. The results also showed that EGCG ameliorated the over-expression of Aβ_1–42_ in the hippocampus and cortex in old rats. In an experiment using senescence-accelerated animal model SAMP10 mice, Pervin et al. [158] found that EGCG and a combination of epigallocatechin and gallic acid, which are hydrolysis products of EGCG, improved the learning ability. These authors also showed that SH-SY5Y cell growth was significantly enhanced by 0.05 μM EGCG together with induction of neurite outgrowth.

### 4.2. Effects of Coffee/CGA on Other NDDs and Healthy Subjects

#### 4.2.1. Human Studies of Effects of Coffee/CGA on other NDD and Healthy Subjects

Although a search on www.clinicaltrials.gov (accessed in September 2020) indicated no ongoing clinical trial to ascertain the efficacy of CGA in the treatment of different neurological diseases, several human studies have suggested its anti-NDD effect. That coffee consumption may protect against cognitive decline is revealed by a population-based cohort study showing that moderate consumption of 3 to 5 cups of coffee per day is associated with a lower risk for cognitive decline unrelated to AD [159].

The result of RCT on 39 healthy older participants showed that compared with the decaffeinated coffee containing CGA and placebo, caffeinated coffee showed a robust positive effect on mood and attention processes [160]. However, the decaffeinated coffee rich in CGA also improved some of these measures compared with regular decaffeinated coffee, suggesting that CGA may contribute to these effects. Another RCT, in which 60 healthy older adults aged ≥50 years were administered 6 g of a decaffeinated green coffee blend or 540 mg CGA or placebo, found that CGA did not improve cognitive function relative to placebo, whereas the decaffeinated green coffee improved sustained attention [161]. Both the green coffee blend and CGA were found to significantly improve symptoms of headache and jitteriness relative to placebo, suggesting that CGA may contribute to the improvements in mood to some extent. The results of an RCT conducted by Saitou et al. [162] on 38 healthy participants who were assigned to either CGA group or placebo group showed that the CGA group had a significant increase in the cognitive performance scores for motor speed, psychomotor speed, and executive function compared with the placebo group, as well as an improvement in the shifting attention test scores. In blood analysis, the CGA group showed increased levels of apolipoprotein A1 and transthyretin, both of which are putative biomarkers for early-stage cognitive decline. 

An intervention study showed that the biomarkers of oxidative damage chosen such as 3-nitrotyrosine were lower in high level consumers of instant coffee compared to those who consumed water, suggesting that the consumption of coffee may protect healthy adults from the oxidative damage [163]. In a clinical study with a total of 2513 participants aged ≥60 years, Dong et al. [164] found that the prevalence of low cognitive performance decreased with increasing intake of total coffee and caffeinated coffee with a nonlinear, L-shaped dose–response relationship. However, no significant association was found between decaffeinated coffee and cognitive performance. 

In a 10-year prospective cohort study, an inverse and J-shaped association was observed between the number of cups of coffee consumed and cognitive decline, with the least cognitive decline for 3 cups/day of coffee. This decline was 4.3 times smaller than that of non-consumers [165]. In a survey of 1445 Italian individuals aged 65–84 years with a 3.5-year median follow-up, habitual consumption of coffee (1–2 cups/day) had a lower rate of MCI (HR: 0.47, CI: 0.211, 1.02 for 1 cup/day or HR: 0.31, CI: 0.13, 0.75 for 1–2 cups/day) compared with those who never or rarely consumed coffee [166]. However, no significant association was found in consumers of >2 cups/day of coffee. 

Thus, several human studies have shown beneficial effects of coffee on cognitive functions, but others not as follows.

A dose–response meta-analysis of 8 prospective studies including 7486 dementia cases found no statistically significant association between coffee consumption and the risk of dementia and no deviations from a linear trend. The RR of dementia per 1 cup/day increment of coffee consumption was 1.01 (CI: 0.98, 1.05) [40]. The result of a mean of 5.3 years of follow-up study found no significant association was found between coffee intake and cognitive decline, although green tea consumption had beneficial effect [167]. The results of a meta-analysis of 20 epidemiological studies from 19 articles, which involved 31,479 participants, showed no association between caffeine intake from coffee or tea and the risk of cognitive disorders [17]. 

#### 4.2.2. Basic Studies of CGA on Other NDDs and Action Mechanism

A number of cell-based and animal experiments have shown beneficial effects of CGA on NDDs. A study by Kim et al. [168] on the protective effect of CGA against oxidative damage found that CGA inhibited H₂O₂-induced apoptotic nuclear condensation in neuronal cells. CGA ameliorated the H₂O₂-induced down-regulation of anti-apoptotic proteins Bcl-2 and Bcl-X(L) with blocking H₂O₂-induced pro-apoptotic cleavage of caspase-3 and pro-poly(ADP-ribose) polymerase (PARP). Similarly, Cho et al. [169] demonstrated CGA’s neuroprotection against H₂O₂-induced oxidative stress in PC12 cells. The CGA-treated cells exhibited less production of ROS, reduction in nuclear condensation and DNA fragmentation as well as inhibited cleavage of PARP and downregulation of Bcl-X(L) and caspase-3. CGA inhibited the H₂O₂-activation of c-Jun *N*-terminal protein kinase and p38 mitogen-activated protein kinase. 

Using cultures of retinal ganglion cells, Nakajima et al. [170] demonstrated that CGA increased cellular protection against oxidative stress induced by l-buthionine-(*S*,*R*)-sulfoximine to deplete glutathione in combination with glutamate to inhibit cystine uptake in cultured retinal ganglion cells. In addition, they noted reduced lipid peroxidation in homogenates of mouse forebrain treated with CGA, suggesting its antioxidative activity in vivo. CGA has also been shown to reduce neuroinflammation resulting from overactive primary microglia in the brain. Shen et al. [171] showed that CGA suppressed TNF-α release and NO production in primary microglia treated with LPS, suggesting that CGA increases the survival of dopaminergic neurons by inhibiting neuroinflammation. These authors also demonstrated that CGA pretreatment attenuated LPS-induced IL-1β and TNF-α release in substantia nigra.

In an experiment, in which rats received middle cerebral artery occlusion to induce ischemia, intraperitoneal injections of CGA reduced dose-dependently infarct volume, sensory-motor functional deficits, brain water content together with reduction in lipid peroxidation and in expression and activities of MMP-2 and MMP-9 [172]. The findings suggest that CGA can reduce blood–brain barrier damage and brain edema, leading to prevention of memory impairment. 

### 4.3. Effects of Wine/RSV on Other NDDs

#### 4.3.1. Human Studies of Effects of Wine/RSV on Other NDDs

Red wine is particularly rich in specific polyphenolic compounds that may affect the biological processes of NDDs, such as RSV, quercetin, myricetin, catechins, tannins, anthocyanidins, and ferulic acid [173]. There is a body of evidence to support the neuroprotective effects of red wine polyphenols including RSV.

A number of human intervention studies provided beneficial effects of RSV on NDDs. In a pooled analysis of 3 RCT, Marx et al. [174] found that RSV had a significant effect on delayed recognition (SMD: 0.39, CI: 0.08, 0.70) and negative mood (SMD: −0.18, CI: −0.31, −0.05), suggesting that RSV might improve cognitive performance. A 34-year follow-up of the prospective population study of women in Sweden found that wine was protective for dementia (HR: 0.6, CI: 0.4, 0.8) and the association was strongest among women who consumed wine only (HR: 0.3, CI: 0.1, 0.8) [175]. These authors suggest that components other than ethanol may be associated with wine’s effect, since a protective effect was not found for the other beverages. In a longitudinal large population-based study on 5033 stroke-free men and women in Norway, the result of 7 years follow-up showed light-to-moderate wine consumption was associated with better performance on cognition tests [176].

A recent comprehensive review by Cicero et al. [60] has discussed effects of RSV on humans based on the previous studies including 9 RCTs. For example, an RCT on 23 healthy overweight older adults that successfully completed 26 weeks of RSV intake (200 mg/day) and 23 participants in the placebo group, found a significant effect of RSV on retention of words. The RSV group showed increases in hippocampal functional connectivity and the serum levels of leptin, and decreases in glycated hemoglobin and body fat [177]. These results suggest that supplementary RSV improves memory performance in older adults. An RCT for 80 post-menopausal women aged 45–85 years showed that consumption of RSV for 14 weeks caused significant improvements in the performance of cognitive tasks in the domain of verbal memory and in overall cognitive performance compared to placebo [178]. A 24-month RCT undertaken in 125 postmenopausal women, aged 45–85 years found that consumption of 75 mg RSV twice-daily for 12 months resulted in a 33% improvement in overall cognitive performance [179]. In addition, RSV improved cerebrovascular function and insulin sensitivity in postmenopausal women. The findings may be translated into a delaying of the cognitive decline due to aging and menopause.

On the other hand, several studies failed to show the RSV’s favorable effect. A systematic review of 10 RCTs found no effect on cognitive performance in 5 studies [174]. For example, an RCT in 22 healthy adults demonstrated that a consumption of 250 and 500 mg of RSV gave no effect on cognitive function, although increases were observed in cerebral blood flow during task performance and deoxyhemoglobin [174,180]. An RCT, in which the RSV group received orally 500 mg RSV once daily with dose escalation by 500-mg increments every 13 weeks and ending with 1000 mg twice daily, showed no effects of RSV treatment on biomarkers including plasma Aβ_42_, CSF Aβ_42_, CSF tau, CSF p-tau 181, hippocampal volume, entorhinal cortex thickness, mental state evaluation or glucose metabolism, suggesting the difficulty in showing RSV’s benefit [181]. These negative results obtained from human studies in spite of the application of much higher amounts of RSV than the content (a few mg/L) in wines [182,183] may reflect a very low bioavailability of RSV. 

#### 4.3.2. Basic Studies of RSV’s Effects on Other NDDs and Action Mechanism

In an experiment using vascular dementia model rats showing decreased cognitive function, increased hippocampal content of MDA, Bax, and caspase‑3, and reduced hippocampal expression of SOD and Bcl‑2, intraperitoneal injection of RSV for 4 weeks caused improvement in these biomarkers [184]. RSV also improved the spatial learning and memory of these model rats. The findings suggest that the RSV’s neuroprotective effects can be closely related to the inhibition of the apoptosis pathway and oxidative stress injury [184]. 

In an experiment using type 2 diabetes model mice, RSV was found to prevent the learning and memory decline by reducing type 2 diabetes-induced hippocampal neuron destruction and synaptic ultrastructural damage and to recover the increased expression levels of inflammatory factors, TNF-α and IL-1β, and oxidative stress-related indicators such as MDA in the model mice [185]. In addition, RSV attenuated the decreased expression of nuclear factor erythroid-2-related factor (Nrf2) and its downstream target genes, heme oxygenase-1 (HO-1), NAD(P)H-quinone oxidoreductase, metallothionein, SOD, and catalase. In an experiment to examine an RSV’s effect on age-induced cognitive impairment, Gocmez et al. [186] found that aged rats showed cognitive impairment in the passive avoidance test, which was improved by the administration of RSV at 50 mg/kg/day. RSV attenuated the increased protein levels of TNF-α and IL-1β induced by aging, suggesting an RSV’s role in preventing cognitive impairment in aged rats by inhibiting the production of inflammatory cytokines.

Based on the previous findings, Bastianetto et al. [187] proposed mechanisms underlying RSV’s actions. RSV’s properties involved are: (1) vasodilatory activity; (2) free radical and ion metal scavenging activities; (3) increasing activity for HO-1 protein levels and phosphorylation of GSK-3β; (4) reducing activity for the expression of COX-2 protein and TNF-α in the substantia nigra and the production/expression of prostaglandins, NO, TNF-α, COX-1/2, and NF-κB; (5) inhibitory activity against the activation of mitogen-activated protein kinase (MAPK) and NF-κB in microglia, inducing activity of the phosphorylation of PKCδ isoform; (6) inhibitory and destabilizing activities of Aβ_1–42_ fibril formation; (7) enhancing activity of the degradation of Aβ via a mechanism that involves proteasome; and (8) promoting activity of the AMPK/SIRT1 signaling pathway. 

In an experiment employing the murine nigrostriatal pathway injury model, which leads to neurological dysfunction or neuron necrosis, Li et al. [188] found that RSV decreased the p-ERK expression and increased the p-JNK expression compared to the vehicle group, but not alter the p38 MAPK proteins around the lesion site. The results also showed that RSV treatment had a neuroprotective and anti-inflammatory effects with the decreased level of IL-1β, TNF-α, and IL-6. RSV up-regulated the protein expression of p-JNK and Bcl-2 and down-regulated the expression of Bax. The findings suggest that RSV attenuates injury-induced neuronal apoptosis and inflammation via activation of c-JNK signaling. Zhao et al. [189] examined the effects of RSV on HFD-induced obesity with postoperative cognitive dysfunction and found that RSV recovered the memory loss caused by HFD along with increasing expression of hippocampal Sirt1 and PGC-1α, leading to attenuation of HFD-induced reduction in the levels of fibronectin type III domain-containing protein-5 and brain-derived neurotrophic factor. 

Cicero et al. [60] summarized mechanistic aspects of RSV’s neuroprotective action. RSV displays: (1) antioxidant activity by scavenging free radicals and metals; (2) protection against NO toxicity; (3) reduction of quinone reductase 2 activity and upregulation of enzymes such as AMPK, GSH, and liver kinase B1 (LKB1); (4) inhibition of expression of pro-inflammatory enzyme such as COX-1, NF-κB activation as well as PGE2, NO, and TNF-α production, and cytokine release; (5) activation of SIRT1; and (6) promotion of Aβ clearance. RSV can also affect multiple signaling pathways involved in cell survival (AMPK, phosphatidylinositol-3-kinase, and Akt), apoptosis (caspase-3/12, Bax, and cytochrome c) and synaptic plasticity (ERK1/2).

### 4.4. CRC’s Effects on Others NDDs and Healthy Subjects

#### 4.4.1. Human Studies of CRC’s Effects on Other NDDs and Healthy Subjects

In a cohort study on 1010 nondemented elderly Asian subjects aged 60–93 years, Ng et al. [190] found that subjects who consumed curry “occasionally” and “often or very often” showed significantly better mental state examination scores than those who “never or rarely” consumed curry. Clinical trials on CRC have confirmed its therapeutic potential in the treatment of various NDDs. A search on www.clinicaltrials.gov (accessed in September 2020) indicated around 18 ongoing clinical trials to ascertain the efficacy of CRC in the treatment of different neurological diseases. Although many clinical trials examining the efficacy of CRC in patients with cognitive decline have been disappointing, more recent studies using improved CRC formulations and more appropriate cohorts have provided encouraging signs [191]. 

Cox et al. [192] examined acute (1- or 3-h post dose) and chronic (1-month duration) effects of CRC intake on cognition, mood and blood biomarkers in an RCT. Benefits on attention and working memory were observed after the acute administration of CRC, and working memory and mood were improved at the 1-month time point. In an RCT, 40 non-demented subjects (age 51–84 years) were randomized to a bioavailable form of CRC or placebo for 18 months, CRC but not placebo improved verbal memory, visual memory, and attention compared with placebo [193]. CRC’s symptom benefits were associated with decreases in amyloid and tau protein accumulation in brain regions modulating mood and memory. Daily consumption of CRC may lead to improved memory and attention in non-demented adults [193].

To confirm the effects of CRC on cognitive functioning and inflammatory state in Schizophrenia, a pilot RCT was conducted [194]. The results showed that CRC improved working memory in patients with schizophrenia and reduced IL-6 levels after 8 weeks. An RCT on 96 participants who ingested either placebo or 1500 mg/day CRC formulation for 12 months, showed a significant time × treatment group interaction for a cognitive assessment test [195]. This interaction was associated with a decline in function of the placebo group at 6 months that was not observed in the CRC-treated group. 

#### 4.4.2. Basic Studies of CRC on Other NDDs and Action Mechanism 

In addition to several human studies which have provided mechanistic aspects of CRC’s beneficial actions as mentioned above, a body of evidence has also been obtained by animal and cell-based studies. A recent review by Voulgaropoulou et al. [78] included 11 animal studies to show CRC’s favorable effects on age-related cognitive dysfunctions. For example, CRC attenuated cognitive deficits of SAMP8 by decreasing the oxidative stress to increase the hippocampal MDA content and by improving the hippocampal expression of phosphorylated calcium/calmodulin-dependent kinase (CaMKK) II and phosphorylated N-methyl-D-aspartate receptor subunit 1 [196]. Dong et al. [197] demonstrated that CRC enhanced non-spatial and spatial memory as well as dentate gyrate cell proliferation in aged rats. 

In experiments using established LPS-treated BV2 microglial cells and a mouse model as neuroinflammation model, Qiao et al. [198] found that CRC was neuroprotective and that CRC activated AMPK in BV2 microglial cells through CaMKK-β and LKB1. Bhat et al. [77] presented a summary of molecular mechanism of CRC’s action of curcumin in different NDDs. CRC can upregulate Nrf2, mitochondrial biogenesis, and dopamine release, while it may downregulate or inhibit Aβ and synuclein aggregation, APP gene expression, β-secretase, oxidative damage, NF-κB activation, MAPK signaling pathway, PPAR-γ, mitochondrial dysfunction, inflammation, apoptosis, and ROS. 

In an elegant study, Dong et al. [197] demonstrated a neurogenesis- and cognition-enhancing potential of 6- and 12-week treatment with CRC in aged rats and revealed CRC-induced gene expression changes in cortex and hippocampus of these rats. The expression of 81 hippocampal genes and 132 cortical genes in rats treated with CRC for 6 weeks were changed by ≥1.5-fold, whereas 55 genes in the hippocampus and 162 genes in the cortex in rats treated for 12-week were changed by 1.5-fold. These genes were related to multiple signal pathways responsible for CRC-stimulation in modulation of neurotransmission and metabolic homeostasis, neuronal development, signal transduction, transport as well as RNA transcription. The results also showed that CRC affected divergent cognition-associated neuronal network in a treatment duration- and tissue-dependent manner.

## 5. Discussion

This review article shows that considerable amounts of human observational and intervention studies have revealed the beneficial effects of consumption of tea, coffee, wine, and curry and their principal polyphenols on NDDs. However, other studies failed to show such effects. The inconsistent results may be due to confounding factors including differences in study design, the method of quantifying consumption, beverage temperature, cigarette smoking, alcohol consumption, and differences in genetic and environmental factors such as race, gender, age, lifestyle, intestinal microbiota, and genetic polymorphisms [1,5,199]. 

In these confounding factors, there are several ones which are difficult to be adjusted. For example, polyphenols can affect intestinal microbiota as described in a recent comprehensive review to show the important roles of polyphenols including those discussed here [200]. However, it appears difficult to adjust the intestinal microbiota of an individual person in epidemiological studies. Furthermore, these foods contain various polyphenols and bioactive compounds which might synergistically contribute to the anti-NDDs’ effects, although it is difficult to adjust such synergistic effects in human epidemiological studies. Well-designed human intervention studies may provide detailed information whether or not dietary polyphenols have benefits on human diseases including NDDs in the future.

**Figure 2:**
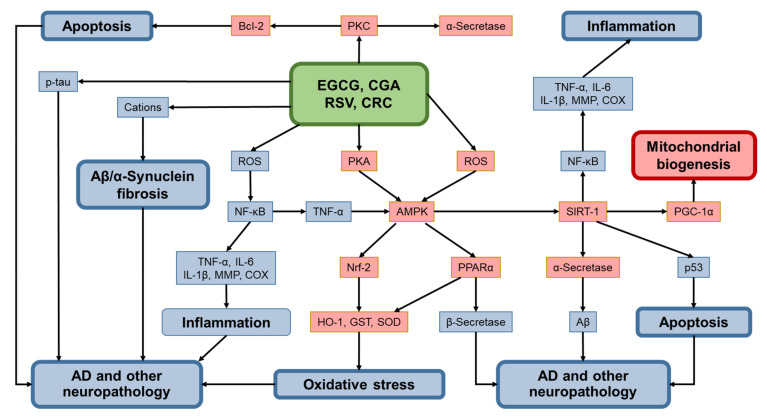
A putative mechanism under which EGCG, CGA, RSV, CRC exert anti-DDs effects. Red-colored boxes represent upregulation/stimulation and blue ones represent downregulation/suppression.

Many animal and cellular experiments and some human studies have provided molecular basis for anti-NDD effects of principal polyphenols present in these foods. Plant polyphenols are involved in defense against ultraviolet radiation and aggression by pathogens and insects [2]. Therefore, one can assume that 4 polyphenols described here exhibit very similar biological activities on NDDs. Many of these effects on NDDs can be explained by molecular events illustrated in Figure 2 which is depicted based on previous data [1,3,5,6,8,23,24,53,198,201,202,203,204]. These polyphenols in common can upregulate or activate AMPK [1,114,129,187,198], SIRT1 [25,129,130,187,198,205], and PGC-1α [23,189,205,206] and downregulate ROS and NF-κB [1,3]. 

Dual property of these polyphenols acting as an anti-oxidant and a pro-oxidant has been well documented [1,3,5]. However, it remains unknown how these polyphenols can activate AMPK, although involvement of ROS, protein kinase A (PKA), CaMKK-β, and LKB1 has been postulated [1,3,207]. In addition, it is not established what can direct for theses polyphenols to act as either an anti-oxidant or a pro-oxidant, although cell-types, available cellular polyphenol concentrations, cellular cation concentrations, and cellular redox state can be important factors as discussed previously [1,3,5]. Additionally, it should be noted that activation of AMPK via downregulation of TNF-α is illustrated in Figure 2 based on the finding by Steinberg et al. [208], but its establishment needs future studies. 

As described above, time-dependent and neuronal tissue-dependent changes in gene expression have been detected in CRC-treated aged rats [197]. A similar research would be helpful to clarify the mechanism of polyphenol’s action on NDDs and other diseases.
